# Supplementary material for: Functional geometry of the cortex encodes dimensions of consciousness
Source: Nat Commun. 2023 Jan 5;14:72. doi: 10.1038/s41467-022-35764-7 (PMC9814511; doi:10.1038/s41467-022-35764-7)
Supplement: Supplementary file 1 — Supplementary Information [file 41467_2022_35764_MOESM1_ESM.pdf]

Supplementary Information

Functional geometry of the cortex encodes dimensions of consciousness

Huang, et al.

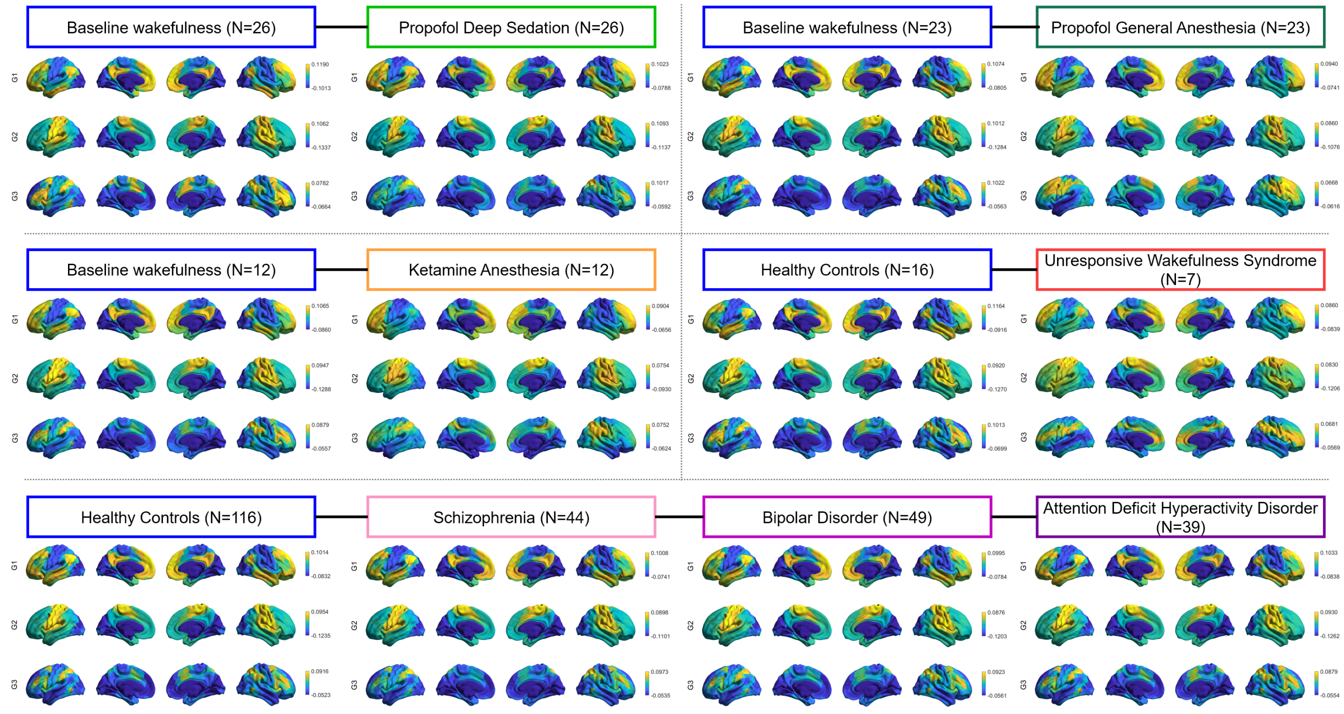

Supplementary Fig. 1 Topographic profiles of the first three cortical gradients across all conditions. Diffusion map embedding was applied on the group averaged functional connectivity matrix in each condition. Gradient eigenvector loading values are mapped onto the cortex along each cortical gradient. G1: Grandient-1; G2: Grandient-2; G3: Grandient-3.

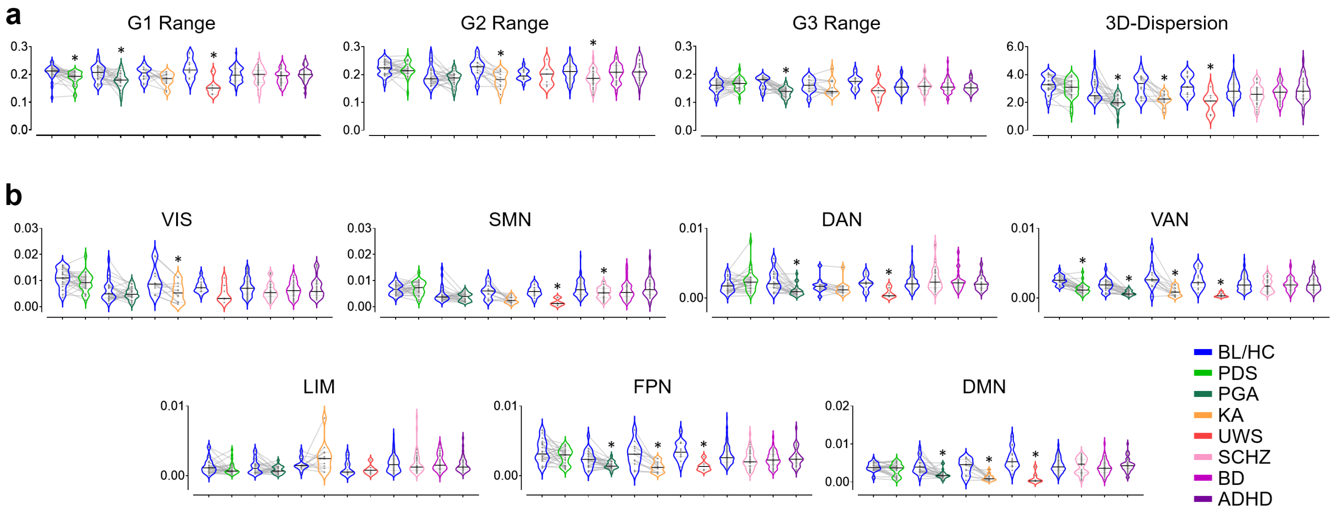

Supplementary Fig. 2 Gradient ranges, 3D-disperssion, and network eccentricity calculated without applying global signal regression during data preprocessing. **a** Gradient ranges and 3D-disperssion across all conditions. **b** Network eccentricity across all conditions. For each measurement, Bayesian Paired Samples T-Tests (two-tailed) were performed for propofol deep sedation (PDS; n=26), propofol general anesthesia (PGA; n=23), and ketamine anesthesia (KA; n=12) against their own baseline conditions (BL; n=26, n=23, n=12, respectively). Bayesian Independent Samples T-Tests (two-tailed) were performed for unresponsive wakefulness syndrome (UWS; n=7), schizophrenia (SCHZ; n=44), bipolar disorder (BD; n=49), and attentional deficit hyperactivity disorder (ADHD; n=39) against their own healthy control groups (HC; n=16, n=116, n=116, n=116, respectively). Asterisk indicates $BF_{10} > 10$. VIS: visual network; SMN: somatomotor network: DAN: dorsal attention network; VAN: ventral attention/salience network; LIM: limbic network; FPN: frontoparietal network; DMN: default-mode network. Detailed statistics are provided in Supplementary Data 2. Source data are provided as a Source Data file.

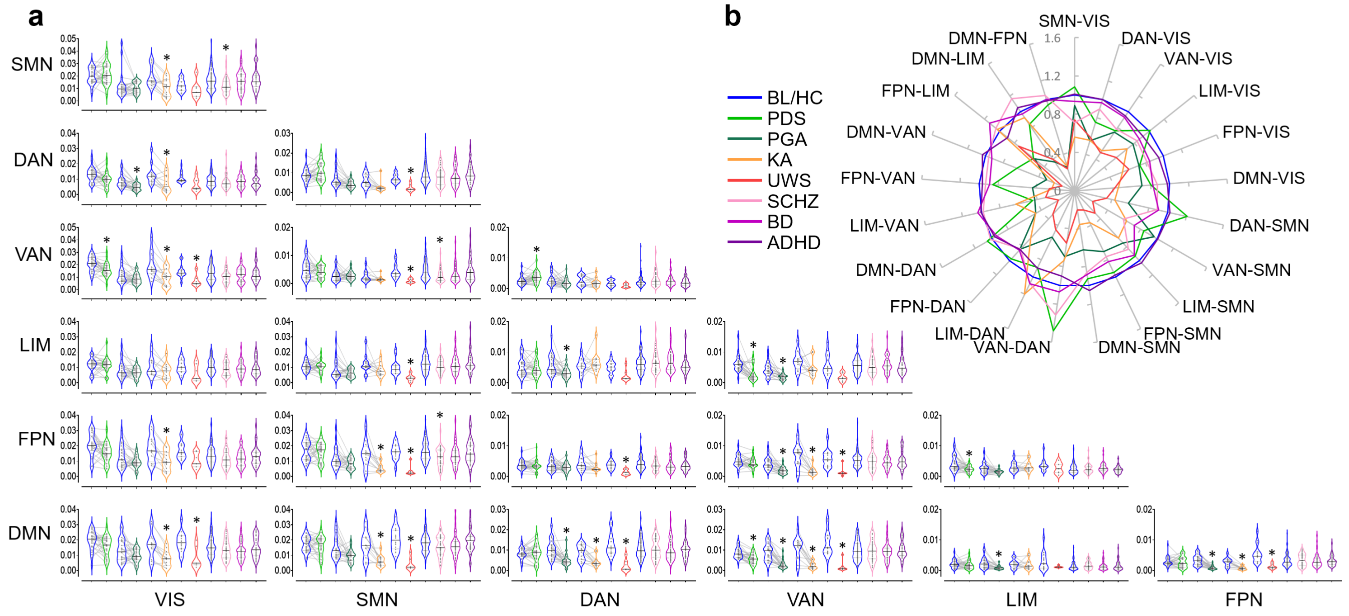

Supplementary Fig. 3 Network distance calculated without applying global signal regression during data preprocessing. **a** Violin plots (median: solid line; quantiles: dash line) of pair-wise network distance, measured by the Euclidean distance between the centroids of two networks in the 3D gradient space, across all conditions. Bayesian Paired Samples T-Tests (two-tailed) were performed for propofol deep sedation (PDS; n=26), propofol general anesthesia (PGA; n=23), and ketamine anesthesia (KA; n=12) against their own baseline conditions (BL; n=26, n=23, n=12, respectively). Bayesian Independent Samples T-Tests (two-tailed) were performed for unresponsive wakefulness syndrome (UWS; n=7), schizophrenia (SCHZ; n=44), bipolar disorder (BD; n=49), and attentional deficit hyperactivity disorder (ADHD; n=39) against their own healthy control groups (HC; n=16, n=116, n=116, n=116, respectively). Asterisk indicates $BF_{10} > 10$. **b** Spider plots displaying mean values of network distance across all conditions. Values for each depressed state of consciousness or psychiatric diagnosis were normalized by dividing the mean of their own baseline (or healthy control) conditions. VIS: visual network; SMN: somatomotor network: DAN: dorsal attention network; VAN: ventral attention/salience network; LIM: limbic network; FPN: frontoparietal network; DMN: default-mode network. Detailed statistics are provided in Supplementary Data 2. Source data are provided as a Source Data file.

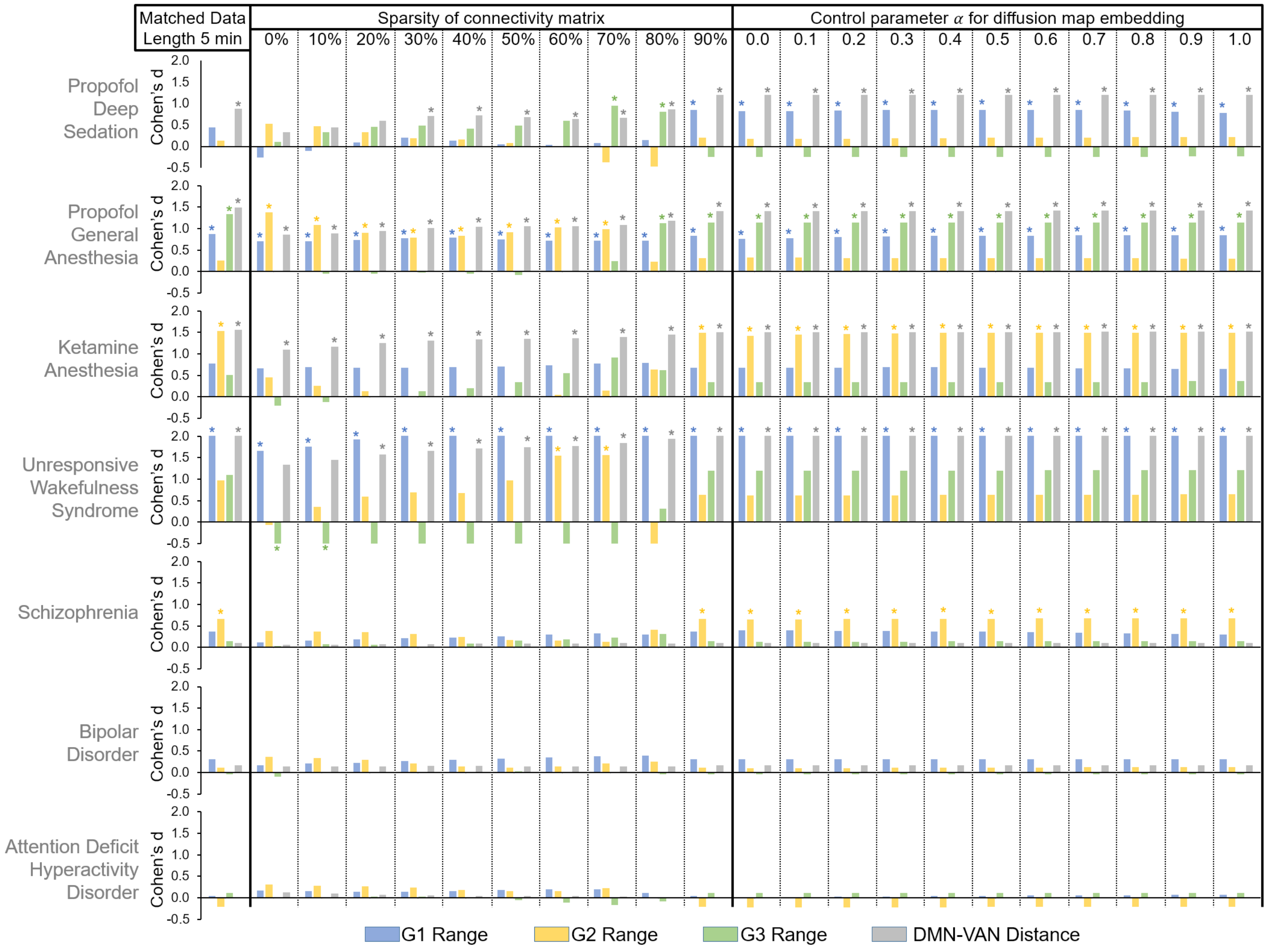

Supplementary Fig. 4 Control analyses of matched data length, varied sparsity and varied parameter α. The original fMRI data were reanalyzed by trimming the data length with a fixed duration of 5 minutes starting from the onset of each scan, varying the sparsity from 0% to 90% (by 10% increments) with parameter α=0.5, and varying the parameter α from 0 to 1 (by 0.1 increments) with sparsity=90%. Bayesian Paired Samples T-Tests (two-tailed) were performed for propofol deep sedation (PDS; n=26), propofol general anesthesia (PGA; n=23), and ketamine anesthesia (KA; n=12) against their own baseline conditions (BL; n=26, n=23, n=12, respectively). Bayesian Independent Samples T-Tests (two-tailed) were performed for unresponsive wakefulness syndrome (UWS; n=7), schizophrenia (SCHZ; n=44), bipolar disorder (BD; n=49), and attentional deficit hyperactivity disorder (ADHD; n=39) against their own healthy control groups (HC; n=16, n=116, n=116, n=116, respectively). Asterisk indicates $BF_{10} > 10$. Bar diagrams represent the Cohen's d derived from the above group comparisons. Detailed statistics are provided in Supplementary Data 3. Source data are provided as a Source Data file.

Excel files are provided for Supplementary Data 1-3:

Supplementary Data 1 Summary of statistics for all measurements (GSR)

Supplementary Data 2 Summary of statistics for all measurements (non-GSR)

Supplementary Data 3 Summary of statistics for control analyses
