# Supplementary material for: Functional geometry of the cortex encodes dimensions of consciousness
Source: Nat Commun. 2023 Jan 5;14:72. doi: 10.1038/s41467-022-35764-7 (PMC9814511; doi:10.1038/s41467-022-35764-7)
Supplement: Supplementary file 3 — Description of Additional Supplementary Files [file 41467_2022_35764_MOESM3_ESM.pdf]

Description of Additional Supplementary Files

Supplementary Data 1. Summary of statistics for all measurements (GSR). Global signal regression was applied during data preprocessing. For each measurement, Bayesian Paired Samples T-Tests (two-tailed) were performed for propofol deep sedation (PDS; n=26), propofol general anesthesia (PGA; n=23), and ketamine anesthesia (KA; n=12) against their own baseline conditions (BL; n=26, n=23, n=12, respectively). Bayesian Independent Samples T-Tests (two-tailed) were performed for unresponsive wakefulness syndrome (UWS; n=7), schizophrenia (SCHZ; n=44), bipolar disorder (BD; n=49), and attentional deficit hyperactivity disorder (ADHD; n=39) against their own healthy control groups (HC; n=16, n=116, n=116, n=116, respectively). Classical Student's T-Tests (paired or independent samples) were also performed for calculating t and p values. Using the Benjamini–Hochberg procedure, p values were false discovery rate–corrected for multiple comparisons for each dataset and thresholded at $\alpha = 0.05$. Assumption checks were performed with Shapiro-Wilk test for normality and Levene's test for homogeneity of variances. For comparisons that violated either of the assumptions, non-parametric statistics are provided, i.e., Bayesian Wilcoxon Signed-Rank Tests for paired samples, and Bayesian Mann-Whitney U Tests for unpaired samples.

Supplementary Data 2. Summary of statistics for all measurements (non-GSR). Global signal regression was not applied during data preprocessing. For each measurement, Bayesian Paired Samples T-Tests (two-tailed) were performed for propofol deep sedation (PDS; n=26), propofol general anesthesia (PGA; n=23), and ketamine anesthesia (KA; n=12) against their own baseline conditions (BL; n=26, n=23, n=12, respectively). Bayesian Independent Samples T-Tests (two-tailed) were performed for unresponsive wakefulness syndrome (UWS; n=7), schizophrenia (SCHZ; n=44), bipolar disorder (BD; n=49), and attentional deficit hyperactivity disorder (ADHD; n=39) against their own healthy control groups (HC; n=16, n=116, n=116, n=116, respectively). Classical Student's T-Tests (paired or independent samples) were also performed for calculating t and p values. Using the Benjamini–Hochberg procedure, p values were false discovery rate–corrected for multiple comparisons for each dataset and thresholded at $\alpha = 0.05$. Assumption checks were performed with Shapiro-Wilk test for normality and Levene's test for homogeneity of variances. For comparisons that violated either of the assumptions, non-parametric statistics are provided, i.e., Bayesian Wilcoxon Signed-Rank Tests for paired samples, and Bayesian Mann-Whitney U Tests for unpaired samples.

Supplementary Data 3. Summary of statistics for control analyses. Control analyses were performed with matched data length, varied sparsity and varied parameter α. The original fMRI data were reanalyzed by trimming the data length with a fixed duration of 5 minutes starting from the onset of each scan, varying the sparsity from 0% to 90% (by 10% increments) with parameter α=0.5, and varying the parameter α from 0 to 1 (by 0.1 increments) with sparsity=90%. Bayesian Paired Samples T-Tests (two-tailed) were performed for propofol deep sedation (PDS; n=26), propofol general anesthesia (PGA; n=23), and ketamine anesthesia (KA; n=12) against their own baseline conditions (BL; n=26, n=23, n=12, respectively). Bayesian Independent Samples T-Tests (two-tailed) were performed for unresponsive wakefulness syndrome (UWS; n=7), schizophrenia (SCHZ; n=44), bipolar disorder (BD; n=49), and attentional deficit hyperactivity disorder (ADHD; n=39) against their own healthy control groups (HC; n=16, n=116, n=116, n=116, respectively). Classical Student's T-Tests (paired or independent samples) were also performed for calculating t and p values. Global signal regression (GSR) was applied during data preprocessing.
