# Supplementary material for: Functional geometry of the cortex encodes dimensions of consciousness
Source: Nat Commun. 2023 Jan 5;14:72. doi: 10.1038/s41467-022-35764-7 (PMC9814511; doi:10.1038/s41467-022-35764-7)
Supplement: Supplementary file 7 — Reporting Summary [file 41467_2022_35764_MOESM7_ESM.pdf]

nature portfolio

Corresponding author(s): Zirui Huang

Last updated by author(s): Oct 10, 2022

Reporting Summary

Nature Portfolio wishes to improve the reproducibility of the work that we publish. This form provides structure for consistency and transparency in reporting. For further information on Nature Portfolio policies, see our Editorial Policies and the Editorial Policy Checklist.

Statistics

For all statistical analyses, confirm that the following items are present in the figure legend, table legend, main text, or Methods section.

n/a	Confirmed	
☐	☒	The exact sample size (n) for each experimental group/condition, given as a discrete number and unit of measurement
☐	☒	A statement on whether measurements were taken from distinct samples or whether the same sample was measured repeatedly
☐	☒	The statistical test(s) used AND whether they are one- or two-sided<br>*Only common tests should be described solely by name; describe more complex techniques in the Methods section.*
☐	☒	A description of all covariates tested
☐	☒	A description of any assumptions or corrections, such as tests of normality and adjustment for multiple comparisons
☐	☒	A full description of the statistical parameters including central tendency (e.g. means) or other basic estimates (e.g. regression coefficient) AND variation (e.g. standard deviation) or associated estimates of uncertainty (e.g. confidence intervals)
☐	☒	For null hypothesis testing, the test statistic (e.g. F, t, r) with confidence intervals, effect sizes, degrees of freedom and P value noted<br>*Give P values as exact values whenever suitable.*
☐	☒	For Bayesian analysis, information on the choice of priors and Markov chain Monte Carlo settings
☒	☐	For hierarchical and complex designs, identification of the appropriate level for tests and full reporting of outcomes
☐	☒	Estimates of effect sizes (e.g. Cohen's d, Pearson's r), indicating how they were calculated

Our web collection on statistics for biologists contains articles on many of the points above.

Software and code

Policy information about availability of computer code

Data collection	The verbal instructions were programmed using E-Prime 3.0 (Psychology Software Tools, Pittsburgh, PA) and delivered via an audiovisual stimulus presentation system designed for an MRI environment. Behavioral responses were measured in mmHg of air pressure during squeezing the rubber ball, using BIOPAC (https://www.biopac.com) MP160 system with AcqKnowledge software (V5.0).
Data analysis	Publicly available software and toolbox used for analyses include AFNI (linux_ubuntu_16_64; http://afni.nimh.nih.gov/), MATLAB R2017b, BrainSpace (https://brainspace.readthedocs.io/en/latest/), and JASP v0.16.3 (https://jasp-stats.org/). Additional custom code is publicly available from Zenodo repository (https://doi.org/10.5281/zenodo.6955280).

For manuscripts utilizing custom algorithms or software that are central to the research but not yet described in published literature, software must be made available to editors and reviewers. We strongly encourage code deposition in a community repository (e.g. GitHub). See the Nature Portfolio guidelines for submitting code & software for further information.

Data

Policy information about availability of data

All manuscripts must include a data availability statement. This statement should provide the following information, where applicable:

- Accession codes, unique identifiers, or web links for publicly available datasets
- A description of any restrictions on data availability
- For clinical datasets or third party data, please ensure that the statement adheres to our policy

The data generated in this study that support our findings have been published (reference 15, reference 21), which are publicly available from Zenodo repository (https://doi.org/10.5281/zenodo.6955280). The psychiatric dataset used in this study is publicly available from OpenfMRI (https://legacy.openfmri.org/dataset/ds000030/). Source data to plot the figures are provided with this paper.

Field-specific reporting

Please select the one below that is the best fit for your research. If you are not sure, read the appropriate sections before making your selection.

- [x] Life sciences
- [ ] Behavioural & social sciences
- [ ] Ecological, evolutionary & environmental sciences

For a reference copy of the document with all sections, see nature.com/documents/nr-reporting-summary-flat.pdf

Life sciences study design

All studies must disclose on these points even when the disclosure is negative.

Sample size	For an fMRI study of cognitive function, Desmond and Glover (referecne 69) reported that about 25 subjects are necessary to achieve 80% power for a 0.5% increase of activity. We also conducted power analysis based on our previous study with graded sedation of propofol (referecne 70). The average Cohen's d as a measure of the effect size was 0.56. For 80% power and α=0.05, n=24 human subjects will be needed. The obtained subjects in Dataset-1 (n=26) and Dataset-2 (n=23) are above or very close to the suggested optimal group size for reliable statistics in functional MRI studies. The number of subjects in Dataset-3 (n=12) and Dataset-4 (n=16, 7) are limited. However, we evaluated the specificity of our results in an independent cohort of 248 subjects consisting of healthy control participants and patients with psychiatric disorders (schizophrenia, bipolar disorder and attention deficit/hyperactive disorder).
Data exclusions	No data were excluded in Dataset-1 or Dataset-3. Dataset-2 originally had 26 subjects. Among them, three subjects had to be excluded from further data analysis because of excessive movements, resulting in 23 subjects. In Dataset-4, 6 out of 13 patients diagnosed as unresponsiveness wakefulness syndrome (UWS) had to be excluded due to cortical distortions, resulting missing values in at least one of 400 cortical areas from the pre-defined brain parcellation scheme. In Dataset-5, the original dataset included 272 subjects encompassing healthy individuals (n=130) and individuals with psychiatric disorders including schizophrenia (SCHZ, n=50), bipolar disorder (BD, n=49), and attention deficit/hyperactivity disorder (ADHD, n=43). Twenty-four subjects were excluded due to lack of T1 images or resting-state data, or the overall head motion range were above 3 mm, or the data had insufficient degree of freedom after band-pass filtering and motion scrubbing. This resulted in 116, 44, 49, and 39 for healthy controls, SCHZ, BD and ADHD in our analysis (248 in total). The exclusion criteria were pre-established.
Replication	One of the key findings, i.e., a degradation of Gradient-1 induced by propofol anesthetics, was first found in Dataset-1 and reproduced independently in Dataset-2.
Randomization	The experiments were not randomized because within-subject design was used in Dataset-1, Dataset-2, and Dataset-3. Dataset-4 and Dataset-5 were collected from independent research sites with different research protocols.
Blinding	The investigators were not blinded to allocation during experiments and outcome assessment.

Reporting for specific materials, systems and methods

We require information from authors about some types of materials, experimental systems and methods used in many studies. Here, indicate whether each material, system or method listed is relevant to your study. If you are not sure if a list item applies to your research, read the appropriate section before selecting a response.

Materials & experimental systems

n/a	Involved in the study	
x		Antibodies
x		Eukaryotic cell lines
x		Palaeontology and archaeology
x		Animals and other organisms
	x	Human research participants
x		Clinical data
x		Dual use research of concern

Methods

n/a	Involved in the study	
x		ChIP-seq
x		Flow cytometry
	x	MRI-based neuroimaging

Human research participants

Policy information about studies involving human research participants

Population characteristics	Dataset-1 included 26 healthy participants (right-handed; male/female: 13/13; age: 19-34 years). All participants were classified as American Society of Anesthesiologists (ASA) physical status I. Dataset-2 included 26 participants (right-handed; male/female: 12/14; age: 27-64 years). They were undergoing an elective trans-sphenoidal approach for pituitary microadenoma resection. The pituitary microadenomas were diagnosed by their size (<10 mm in diameter without growing out of the sellar region) based on radiological examinations and plasma endocrinal parameters. The participants were classified as ASA physical status I or II. Dataset-3 included 12 participants (right-handed; male/female: 7/5; age: 32-66 years). They were undergoing an elective trans-sphenoidal approach for pituitary microadenoma resection, and were classified as ASA physical status I or II. Dataset-4 included 16 healthy controls (male/female: 8/8; age: 23-65 years) and 21 patients (male/female: 18/3; age: 8-78 years) with disorders of consciousness. The patients were assessed using the Coma Recovery Scale-Revised (CRS-R) on the day of fMRI scanning. Of those assessed, 13 patients were diagnosed as unresponsiveness wakefulness syndrome, and 8 were diagnosed as minimally conscious state. The detailed information of Dataset-5 (psychiatric cohort) can be found in a published study by Poldrack et al., 2016, Scientific Data.
Recruitment	Dataset 1: Healthy participants were recruited by listing on UMClinicalStudies.org and by postings at area colleges and community groups in Ann Arbor. Interested volunteers called the phone number of a designated recruiter for an initial phone screening. The initial phone screening consisted of questionnaires related to medical history, demographic information, handedness, inclusion and exclusion criteria and procedure standard MRI screening questionnaire. The participants completed the questionnaires, which were reviewed by the study team. The health status was confirmed by the attending anesthesiologist before the study on site. After the eligibility was confirmed by the study team, the one-time research study session was scheduled. None of the participants had psychiatric or neurological disorders (or a history thereof) in the study.<br><br>Dataset-2, Dataset-3 and Dataset-4: The online recruitment system at Huashan Hospital helped to recruit all healthy participants and patients. For Dataset-2 and Dataset-3, the participants were screened using a study-specific screening form contain medical history and demographic information. They were classified as American Society of Anesthesiologists physical status I or II, with no history of craniotomy, cerebral neuropathy, vital organ dysfunction or administration of neuropsychiatric drugs. They had no contraindication for an MRI examination, such as vascular clips or metallic implants. For Dataset-4, the patients with disorders of consciousness were selected as a convenience sample. For each patient, clinical examination was repeatedly performed using standardized CRS-R assessments. None of the healthy controls had a history of neurological or psychiatric disorders, nor were they taking any kind of medication.<br><br>Dataset-5: Healthy adults were recruited by community advertisements from the Los Angeles area. Participants with adult ADHD, bipolar disorder, and schizophrenia were recruited using a patient-oriented strategy involving outreach to local clinics and online portals (separate from the methods used to recruit healthy volunteers). More detailed information of recruitment can be found in a published study by Poldrack et al., 2016, Scientific Data.<br><br>For all datasets, informed consent was obtained from all participants (or patients' legal representatives in Dataset-4), and they were compensated for participation after the experiment.<br><br>We do not expect any resulting potential self-selection bias.
Ethics oversight	University of Michigan Institutional Review Board, Institutional Review Board of Huashan Hospital at Fudan University, and Institutional Review Boards at UCLA and the Los Angeles County Department of Mental Health.

Note that full information on the approval of the study protocol must also be provided in the manuscript.

Magnetic resonance imaging

Experimental design

Design type	Resting state and task design (event-related).
Design specifications	Dataset-1: Four task fMRI runs were conducted including 15-min wakeful baseline, during (30-min) and after (30-min) propofol infusion, and another 15-min recovery baseline. Behavioral responsiveness was assessed by hand-squeezing a rubber ball, which defined the periods of propofol deep sedation (i.e., loss of behavioral responsiveness). Behavioral responses were measured in mmHg of air pressure using BIOPAC (https://www.biopac.com) MP160 system with AcqKnowledge software (V5.0). Verbal instructions were programmed using E-Prime 3.0 (Psychology Software Tools, Pittsburgh, PA) and delivered via an MRI-compatible audiovisual stimulus presentation system. More details about experimental design can be found in our previous publication (reference 61).<br>Dataset-2: Three 8-min resting-state fMRI scans were conducted including wakeful baseline, propofol light sedation, and propofol general anesthesia.<br>Dataset-3: For the entire experiment, fMRI scanning continued for about one hour, ranging between 44 minutes and 62 minutes. A 10-min conscious baseline was first acquired, except for two participants in which baseline condition was for 6 and 11 minutes. After that, 0.05mg/kg/min of ketamine was infused for 10 minutes (0.5mg/kg in total), followed by 0.1 mg/kg/min for another 10 minutes (1.0mg/kg in total; except for two participants who received 0.1mg/kg/min infusion for 10 minutes). Afterwards, the infusion of ketamine was discontinued, and participants spontaneously regained responsiveness. Behavioral responsiveness was assessed during the fMRI scan. The verbal instruction "press the button" was programmed to play every 30 seconds using E-Prime 2.0 (Psychology Software Tools, Pittsburgh, PA) and delivered via an MRI-compatible audiovisual stimulus presentation system. Participants were instructed to press a

button with their right index finger. The period of loss of behavioral responsiveness, defined as ketamine anesthesia, was determined by comparing the timing of verbal instruction and actual responsiveness during and after ketamine infusion. The fMRI data length of ketamine anesthesia was 18.2±7.6 minutes across participants.
Dataset-4: The resting state fMRI scan lasted 400 seconds.
Dataset-5: The resting state fMRI scan lasted 304 seconds. More detailed information can be found in Poldrack et al. 2016, Scientific Data.

Behavioral performance measures

Dataset-1: Behavioral responses were measured in mmHg of air pressure during squeezing the rubber ball, using BIOPAC (https://www.biopac.com) MP160 system with AcqKnowledge software (V5.0). By comparing the timing of ''action'' instructions and the actual motor response during and after propofol infusion, the periods during which a subject retained responsiveness (PreLOR), loss of responsiveness (LOR), and recovery of responsiveness (ROR) were determined. The offset of PreLOR, onset of LOR, offset of LOR, and onset of ROR were defined as the times of the last successful response of squeezing, the first failure to squeeze, the last failure to squeeze, and the first successful response of squeezing after LOR, respectively.
Dataset-2: Behavioral responsiveness was assessed by the Ramsay scale (Ramsay et al., 1974). A participant was considered fully conscious (Ramsay 1-2) if they responded clearly and strongly to a verbal command ("strongly squeeze my hand!"), or considered mildly sedated if the response was clear but slow (Ramsay 3-4), or considered deeply sedated or anesthetized if there was no response (Ramsay 5–6). The Ramsay scale verbal command was repeated twice for each assessment.
Dataset-3: Behavioral responsiveness was assessed during the fMRI scan. The verbal instruction "press the button" was programmed to play every 30 seconds using E-Prime 2.0 (Psychology Software Tools, Pittsburgh, PA) and delivered via an MRI-compatible audiovisual stimulus presentation system. Participants were instructed to press a button with their right index finger. The period of loss of behavioral responsiveness, defined as ketamine anesthesia, was determined by comparing the timing of verbal instruction and actual responsiveness during and after ketamine infusion.
Dataset-4: The patients were assessed using the Coma Recovery Scale-Revised (CRS-R) (Giacino et al., 2004) on the day of fMRI scanning.
Dataset-5: Enrolled participants completed extensive neuropsychological testing. The list of tests performed during the behavioral session is presented in Poldrack et al. 2016, Scientific Data.

Acquisition

Imaging type(s)

Functional

Field strength

3T

Sequence & imaging parameters

Dataset-1: A 3T Philips scanner with a standard 32-channel transmit/receive head coil. A gradient-echo EPI pulse sequence was used to acquire functional images of the whole brain with the following parameters: 28 slices, TR/TE=800/25ms by multiband acquisition, MB factor=4, slice thickness=4mm, in-plane resolution=3.4×3.4mm; FOV=220mm, flip angle=76°, image matrix: 64×64. Six participants were scanned with slightly different parameters before MRI hardware upgradation (21 slices, TR/TE=800/25ms, MB factor=3, slice thickness=6mm).
Dataset-2 and Dataset-3: A Siemens 3T scanner (Siemens MAGNETOM, Germany) with a standard 8-channel head coil was used to acquire gradient-echo EPI images of the whole brain with the following parameters: 33 slices, TR/TE=2000/30ms, slice thickness=5mm, FOV=210mm, flip angle=90°, image matrix: 64×64.
Dataset 4: A Siemens 3T scanner (Siemens MAGNETOM, Germany) with a standard 8-channel head coil was used to acquire gradient-echo EPI images of the whole brain with the following parameters: 33 slices, TR/TE=2000/35ms, slice thickness=4mm, field of view=256 mm, flip angle=90°, image matrix: 64×64.
Dataset 5: Functional MRI data were collected using a T2*-weighted echoplanar imaging (EPI) sequence with the following parameters: 34 slices, TR/TE=2000/30 ms, slice thickness=4 mm, FOV=192 mm, flip angle=90°, matrix 64×64.

Area of acquisition

Whole brain

Diffusion MRI ☐ Used ☒ Not used

Preprocessing

Preprocessing software

Standardized methods using AFNI (linux_ubuntu_16_64; http://afni.nimh.nih.gov/).

Normalization

AFNI function: @auto_tlrc; a script to transform an antomical dataset to align with standard space template.

Normalization template

Talaraich stereotactic space (TT_N27).

Noise and artifact removal

Using AFNI's function 3dTproject, the time-censored data were band-pass filtered to 0.01 – 0.1 Hz. At the same time, various undesired components (e.g., physiological estimates, motion parameters) were removed via linear regression. The undesired components included linear and nonlinear drift, time series of head motion and its temporal derivative, and mean time series from the white matter and cerebrospinal fluid.

Volume censoring

Frame-wise displacement (FD) of head motion was calculated using frame-wise Euclidean Norm (square root of the sum squares) of the six-dimension motion derivatives. A frame and its each previous frame were excluded if the given frame's derivative value has a Euclidean Norm above FD=0.4 mm.

Statistical modeling & inference

Model type and settings

Based on a well-established brain parcellation scheme (reference 26), the fMRI time courses were extracted from 400 pre-

defined cortical areas (reference 67) after preprocessing. For each participant and condition, a 400x400 connectivity matrix was constructed using Pearson correlation. The group-average (per condition) was calculated by averaging these individual connectivity matrices. Cortical gradients were computed using the BrainSpace toolbox as implemented in MATLAB R2017b. We z-transformed and thresholded the connectivity matrix at the sparsity of 90%, i.e., leaving only the top 10% of weighted connections per row, and calculated a normalized cosine angle affinity matrix that captures the similarity of connectivity profiles between cortical areas. Using a diffusion map embedding algorithm, we identified gradient components, which estimated the low-dimensional embedding from the high-dimensional connectivity matrix. The algorithm is controlled by parameters alpha and t, where alpha controls the influence of the density of sampling points on the manifold and t controls the scale of eigenvalues of the diffusion operator. We fixed alpha at 0.5 and t at 0, preserving global relations between data points in the embedded space. Based on Procrustes rotation, group-level gradient solutions were aligned to a subsample of the HCP dataset (n = 217) available in Brainspace toolbox. We then calculated individual-level gradients for each condition using identical parameters. In order to depict the cortical gradient organization at the network level, the gradient eigenvector loading values were extracted from seven pre-defined functional networks.

We calculated a set of measures to quantify the range of each gradient, global dispersion, network eccentricity, and distance between functional networks in a three-dimensional gradient space. These measures were calculated for each participant within the individualized, aligned gradient space: (1) Numerical range of each gradient was calculated as the distance from the minimum to the maximum gradient eigenvector values, indicating a segregation (i.e., different connectivity profile) of the gradient extremes; (2) Global dispersion was quantified as sum squared Euclidean distance of all regions to the global centroid within the 3D gradient space. A small dispersion value indicates that the functional connectivity profiles across regions have a low differentiation along three gradients; (3) Network eccentricity was calculated as the squared Euclidean distance between a given network centroid and the global centroid; (4) Between-network distance was calculated as the squared Euclidean distance between network centroids. These multi-dimensional distance metrics reflect the similarity of connectivity profiles between cortical areas across multiple axes of differentiation.

Effect(s) tested

Using Bayesian statistics as implemented in JASP (v0.16.3; https://jasp-stats.org/) with default effect size priors, Cauchy scale 0.707, the distance metrics (i.e., gradient range, global dispersion, network eccentricity, network distance) were compared between baseline condition and a given depressed state of consciousness or psychiatric diagnosis. Bayesian Paired Samples T-Tests (two-tailed) were performed for propofol deep sedation, propofol general anesthesia, and ketamine anesthesia against their own baseline conditions. Bayesian Independent Samples T-Tests (two-tailed) were performed for unresponsive wakefulness syndrome, schizophrenia, bipolar disorder, and attentional deficit hyperactivity disorder against their own healthy control groups.

Classical Student's T-Tests (paired or independent samples) were also performed for calculating t and p values. Assumption checks were performed with Shapiro-Wilk test for normality and Levene's test for homogeneity of variances. While the majority of comparisons satisfied both data normality and equal variances, there were some cases that violated one of the two. We therefore performed non-parametric tests for those cases. Specifically, Wilcoxon signed-rank tests were applied to paired samples, and Mann-Whitney U tests were applied to unpaired samples.

Specify type of analysis: ☐ Whole brain ☒ ROI-based ☐ Both

Anatomical location(s): Based on a well-established brain parcellation scheme (reference 26) with 400 cortical areas (reference 67).

Statistic type for inference (See Eklund et al. 2016)

N/A

Correction

Due to the numerous statistical tests performed on different measures across datasets, we sought to minimize potential false inferences by excluding anecdotal (BF10=1-3) and moderate (BF10=3-10) evidence, while collecting strong (BF10=10-30), very strong (BF10=30-100), and decisive (BF10=100) evidence for (H1: there is effect) or against (H0: there is no effect) either hypothesis. Regardless, the strength of the evidence can be judged directly from the numerical value of BF10 reported in Supplementary Data 1. T and p values derived from Classical Student's T-Tests were also reported to complement Bayesian statistics. Using the Benjamini–Hochberg procedure, results were false discovery rate–corrected (FDR-corrected) for multiple comparisons for each dataset and thresholded at $\alpha = 0.05$.

Models & analysis

n/a	Involved in the study
☐	☒ Functional and/or effective connectivity
☒	☐ Graph analysis
☒	☐ Multivariate modeling or predictive analysis

Functional and/or effective connectivity

Pearson correlation
